# Clinical Impact of Neutrophil Variation on COVID-19 Complications

**DOI:** 10.3390/diagnostics15040457

**Published:** 2025-02-13

**Authors:** Khadija El Azhary, Bouchra Ghazi, Fadila Kouhen, Jalila El Bakkouri, Hasna Chamlal, Adil El Ghanmi, Abdallah Badou

**Affiliations:** 1 Laboratory of Immunogenetics and Human Pathologies, Faculty of Medicine and Pharmacy, Hassan II University of Casablanca, Casablanca 20250, Morocco; khadija.elazhary6m@gmail.com; 2 Immunopathology-Immunotherapy-Immunomonitoring Laboratory, Faculty of Medicine, Mohammed VI University of Health and Sciences (UM6SS), Casablanca 82403, Morocco; bghazi@um6ss.ma (B.G.); jelbakkouri@um6ss.ma (J.E.B.); aelghanmi@um6ss.ma (A.E.G.); 3 Department of Gynecology and Obstetrics, Mohammed VI International University Hospital, Bouskoura 27182, Morocco; 4 Laboratory of Neurosciences and Oncogenetics, Neurooncology and Oncogenetic Team, Faculty of Medicine, Mohammed VI University of Health and Sciences (UM6SS), Casablanca 82403, Morocco; fkouhen@um6ss.ma; 5 Department of Radiotherapy, International University Hospital Cheikh Khalifa, Casablanca 82403, Morocco; 6 Cheikh Khalifa International University Hospital, Mohammed VI University of Health and Sciences (UM6SS), Casablanca 82403, Morocco; 7 Computer Science and Systems Laboratory (LIS), Faculty of Sciences Ain Chock, Hassan II University of Casablanca, Casablanca 20250, Morocco; hasna.chamlal@univh2c.ma

**Keywords:** neutrophil, SARS-CoV-2, complication, mortality, prediction, patients with COVID-19

## Abstract

**Background/Objectives**: Corona virus disease 2019 (COVID-19) poses a threat to global public health. The early identification of critical cases is crucial to providing timely treatment to patients. Here, we investigated whether the neutrophil levels could predict COVID-19 complications. **Methods**: We performed a retrospective study of patients with COVID-19, admitted to the Cheikh Khalifa International University Hospital, Casablanca, Morocco. Laboratory test results collected upon admission and during hospitalization were analyzed based on clinical information. **Results**: Our study revealed that a rise in neutrophil “PNN” levels was associated with respiratory deterioration and intubation. They were positively correlated with the procalcitonin and C-reactive protein levels. Interestingly, PNN (polynuclear neutrophil) levels on day 5 proved to be a better predictor of intubation, acute respiratory distress syndrome (ARDS), and mortality than the initial PNN counts, C-reactive protein, or procalcitonin. Moreover, binary logistic regression with stratified PNN-day 5 data revealed that a PNN level on day 5 > 7.7 (109/L) was an independent risk factor for mortality and ARDS. Finally, the PNN levels on day 5 and proinflammatory cytokine IL-6 were positively correlated. **Conclusions**: Our data showed that neutrophilia proved to be an excellent predictor of complications and mortality during hospitalization and could be used to improve the management of patients with COVID-19.

## 1. Introduction

Coronavirus disease 2019 (COVID-19) is a systemic disease causing multiple-organ damage, with the lungs as the main target organs [1]. It is a virus-induced respiratory disease that was first identified in Wuhan in December 2019. The causative virus was named Severe Acute Respiratory Syndrome Coronavirus 2 (SARS-CoV-2). The disease has since rapidly spread to become a worldwide pandemic. As of the 27th of August 2023, 770 million confirmed cases had been reported worldwide, causing over 6.9 million deaths (COVID-19 Weekly Epidemiological Update (WHO)). At present, COVID-19 lacks specific effective drugs. Most commonly, patients with COVID-19 have mild symptoms and good prognosis, while a proportion of patients exhibit severe and critical conditions. These are difficult to treat and show rapid deterioration with or without acute respiratory distress syndrome (ARDS) triggered by a cytokine storm [2,3]. These patients have a poor survival rate and often require intensive medical resource utilization [4,5]. The early identification of patients at risk of severe complications of COVID-19 is of clinical importance. Studies have shown that the progression and prognosis of COVID-19 are related to the body’s immune status and excessive inflammatory response [6,7]. It has been reported that patients with COVID-19 exhibit abnormal results related to the number of immune cells measured during routine peripheral blood tests [8,9]. A decrease in lymphocytes and an increase in the neutrophil–lymphocyte ratio (NLR) are the most obvious abnormalities [10,11]. Previous studies suggested that neutrophil counts are significantly higher for patients presenting with severe forms of the disease [8,12]. Neutrophil infiltration has also been detected upon autopsies [13]. Clarifying the early immunological alterations associated with mild versus severe COVID-19 may not only offer therapeutically actionable targets but also enable the identification of cases at highest risk for clinical deterioration and death. The development of an effective clinical decision-making tool rooted in immunological monitoring has the potential of optimizing patient care and resource utilization.

In this study, we analyzed the clinical and biological characteristics, mainly immunological features, of patients with COVID-19 and investigated the ability of neutrophils to predict, at different stages of infection, the severity and complication of this disease in comparison with other biomarkers.

## 2. Materials and Methods

### 2.1. Study Design and Population

We performed a retrospective study of patients with COVID-19, admitted to the Cheikh Khalifa International University Hospital. We excluded patients less than 15 years old and pregnant women. This study was approved by the institutional ethics board of the University Mohammed VI of Health and Sciences. No patient consent was required as the study included only deidentified information, in accordance with national law.

Patients were classified using two different classifications. In the first classification scheme, we classified the disease severity according to the WHO interim guidance for clinical classification. Non-severe patients (mild “Symptomatic patients (exhibiting fever, cough, fatigue, anorexia, shortness of breath, myalgias) without evidence of viral pneumonia or hypoxia.”) and moderate “patients with clinical signs of pneumonia (fever, cough, dyspnea, fast breathing) but no signs of severe pneumonia, including SpO2 ≥ 90% on room air”, i.e., the asymptomatic group) were designated as “Mild–Moderate”. However, severe patients were those in critical or severe conditions, which required advanced respiratory support. This group was designated as “Severe–Critical”. In the second classification scheme, patients were designated either as “ICU” patients (patients who required care in the intensive care unit, “ICU”, at any point during hospitalization) or “Non-ICU” (patients who did not require ICU care during their hospitalization).

### 2.2. Data Collection

Data were retrospectively collected by reviewing electronic medical records. Patient demographic data included age, sex, past medical history, and usual treatment. Clinical information included date of first symptoms, first positive SARS-CoV-2 results, admission, requirement for oxygen, intubation, hospital stay, intubation duration, admission ward (ICU or not), comorbidities, including hypertension, diabetes, cardiovascular disease, respiratory disease, and other diseases, clinical symptoms including fever, respiratory symptoms, and general symptoms, and clinical outcomes including complication and death. Laboratory test results collected upon admission and during hospitalization included C-reactive protein, procalcitonin, and complete blood count, including neutrophils, lymphocytes, monocytes, and eosinophils.

### 2.3. Statistical Analysis

Descriptive statistics were reported as the mean ± SD or median with a range for the continuous variables and as frequencies with percentages for the categorical variables. The chi-square test was used for comparisons of binary variables. The Mann–Whitney U test was used for the two-group comparison of continuous variables. The Kruskal–Wallis test with Dunn’s multiple comparison was used for the comparison of continuous variables between at least 3 groups. The Spearman rank correlation coefficient was calculated to determine the correlation between two variables. For the prediction analysis, a receiver operating characteristic (ROC) curve analysis was performed, and the difference in the area under the curve (AUC) was tested. Logistic regression analysis with the enter or forward selection method was used to identify risk factors for mortality and ARDS. To address the fact that not all covariates had been registered for all patients, missing values were imputed using multiple imputation (five imputations). After the imputation of the missing values, we assessed neutrophil counts, polynuclear neutrophils on day 5 (PNN-day 5), neutrophil-to-lymphocyte ratio, C-reactive protein, procalcitonin, creatinine, hemoglobin, and age for the prediction of in-hospital mortality and ARDS in our cohort. A *p*-value of <0.05 was considered statistically significant. Analyses were performed using the IBM SPSS Statistics 25 statistical package and GraphPad Prism version 6.01.

## 3. Results

### 3.1. Patient Characteristics According to Disease Severity

We performed a retrospective study including 119 patients presenting with COVID-19. We divided our cohort into two categories, according to the clinical classification of the WHO interim guidance. Non-severe patients were designated as “Mild–Moderate” (*n* = 83), while patients in critical or severe conditions were designated as “Severe–Critical” (*n* = 36). As shown in Table 1, compared to the non-severe cases, the patients with severe COVID-19 were significantly older (mean age, 66 vs. 44 years; *p*-value < 0.001), predominantly male (80.6% vs. 44.6%; *p*-value < 0.001), and characterized by higher rates of clinical symptoms than non-severe cases, including asthenia (55.6% vs. 2.4%; *p*-value < 0.001), fever (68.8% vs. 36.1%; *p*-value = 0.002) and dyspnea (66.7% vs. 18.1%; *p*-value < 0.001). They also exhibited a higher proportion of comorbidities including diabetes (27.8% vs. 8.4%; *p*-value = 0.006) and cardiovascular diseases (30.6% vs. 3.6%; *p*-value < 0.001).

### 3.2. Immune Blood Cell Involvement in COVID-19

**Figure 1:**
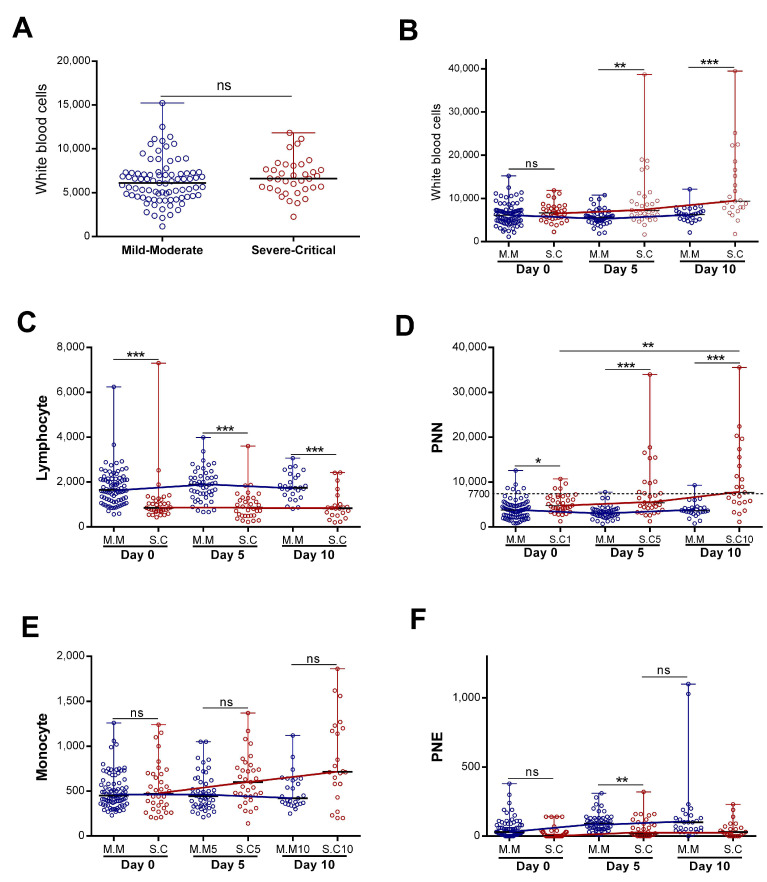
**Evolution of blood immune cell counts depending on COVID-19 severity:** (**A**) initial white blood cells counts (*n* = 83 vs. 36) and(**B**) evolution of white blood cells (*n* = day 0 (83 vs. 36), day 5 (50 vs. 33), and day 10 (27 vs. 23)), (**C**) lymphocyte (*n* = day 0 (83 vs. 35), day 5 (50 vs. 32), and day 10 (27 vs. 22)), (**D**) neutrophil “PNN” (*n* = day 0 (82 vs. 36), day 5 (49 vs. 33), and day 10 (27 vs. 23)), (**E**) monocyte (*n* = day 0 (82 vs. 35), day 5 (50 vs. 33), and day 10 (27 vs. 22)), and (**F**) eosinophil “PNE” counts (*n* = day 0 (83 vs. 36), day 5 (50 vs. 33), and day 10 (27 vs. 22)) during the first 10 days of hospitalization in patients grouped into the “Mild–Moderate”(“M.M”) and “Severe–Critical”(“S.C”) groups. Data are represented as the median with range. Mann–Whitney U, Kruskal–Wallis, and Dunn’s multiple-comparison tests were performed. * *p* < 0.05. ** *p* < 0.01 and *** *p*< 0.001. ns, non-significant.

The immune system could be a good predictor of disease progression. We decided to study the dynamics of white immune cells in two groups of patients with COVID-19 (severe “Severe–Critical” and non-severe “Mild–Moderate”). We found that, upon patients’ admission, there were no statistically significant differences in the total white blood cell (WBC) counts between patients presenting with severe and non-severe disease (Figure 1A). However, when we assessed the WBC numbers as a function of disease evolution, we found that, in patients classified as not nsevere, the number of WBC remained constant during the first ten days of hospitalization (Figure 1B), with no statistically significant differences. On the other hand, the WBC counts increased significantly in the “Severe–Critical” group of patients (*p* = 0.001) (Figure 1B). We then decided to evaluate the evolution, over time, of each cell type separately.

Our study revealed a statistically significant difference in neutrophil and lymphocyte counts during hospitalization in patients presenting with severe disease compared to non-severe disease (Figure 1C,D). The lymphocyte count appeared statistically lower in the severe group. However, the lymphocyte count remained stable during the first ten days of hospitalization in both groups (with no statistically significant differences). The neutrophil profile seemed closely similar to that observed in WBCs (Figure 1B,D). Indeed, we detected a statistically significant increase for patients with severe disease (*p* = 0.017) relative to non-severe disease, and the neutrophil count made it possible to significantly distinguish the two groups (severe “S.C” and non-severe “M.M”) (Figure 1D). With regard to the monocytes, we observed a statistically significant increase in the severe group during hospitalization (*p* = 0.02). The monocyte count did not make it possible to distinguish disease severity (Figure 1E).

**Figure 2:**
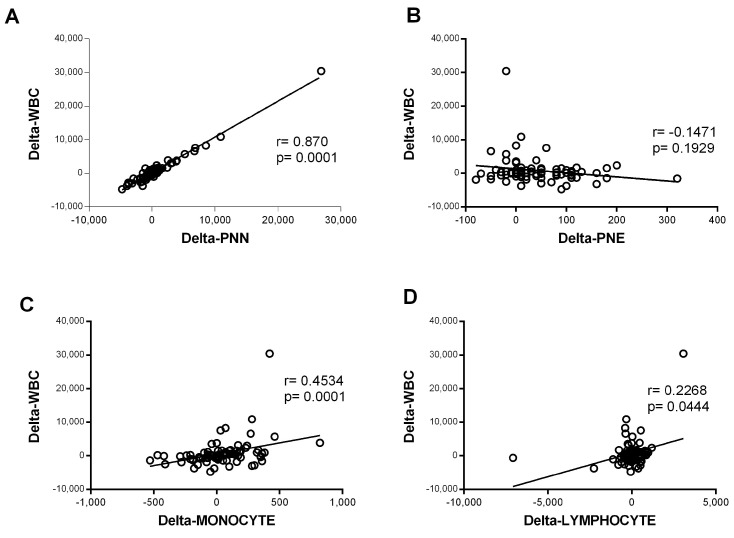
**Correlation of variation in global white blood cells count and variation in the count of each cell type.** The variation was defined as the change in cell counts obtained between day 0 and day 5 of admission (delta = day 5 − day 0). Spearman rank correlation was performed for the white blood cells (WBCs) and (**A**) neutrophils (PNNs) (*n* = 80), (**B**) eosinophils (PNEs) (*n* = 81), (**C**) monocytes (*n* = 82), and (**D**) lymphocytes (*n* = 82).

In contrast, the eosinophil counts increased significantly during hospitalization in the mild–moderate and severe–critical groups (*p* < 0.0001 for M.M and *p* = 0.021 for S.C). The magnitude of the increase was greater in the mild group (Figure 1F). The variation in neutrophil counts was similar to that observed for all WBCs, suggesting that the observed WBC profile was probably due to the evolution of the neutrophil counts. Consistently, further analysis showed a strong correlation between variation in white blood cell counts and variation in neutrophil counts (r = 0.87 and *p* < 0.0001) (Figure 2). Altogether, these observations strongly suggested that neutrophils were the main subpopulation responsible for the WBC increase in patients with COVID-19 presenting with severe–critical disease.

### 3.3. High Neutrophil Counts Significantly Associated with Disease Severity and Inflammatory Biomarker Levels

**Figure 3:**
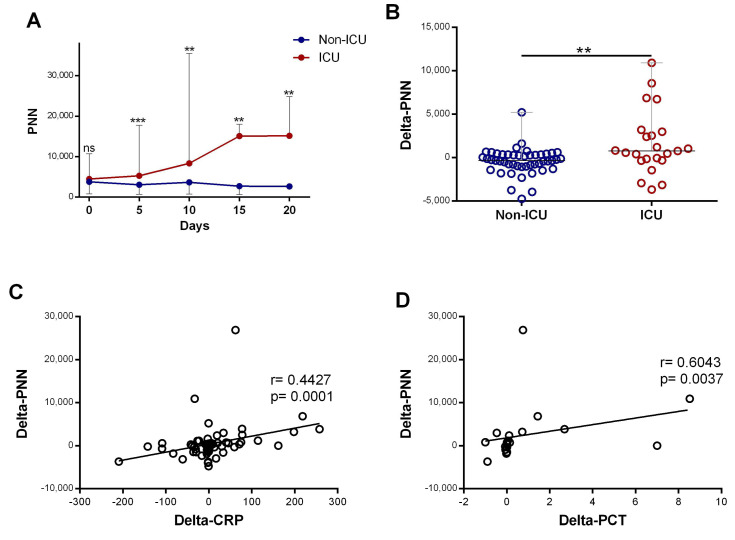
**The increase in neutrophil counts associated with disease severity and the rise in inflammatory biomarkers**: (**A**) evolution of neutrophil(“PNN”) (*n* = day 0 (82 vs. 25), day 5 (49 vs. 23), day 10 (27 vs. 18), day 1 ((9 vs. 8), and day 20 (6 vs. 5)) count during the first 20 days of hospitalization in patients grouped into ICU and non-ICU categories; (**B**) variation in PNN (delta-PNN = PNN-day 5 − PNN-day 0) in ICU (*n* = 23) and non-ICU (*n* = 47) patients; and (**C**) correlation of variation in PNN (delta-PNN, *n* = 80) and variation in CRP (delta-CRP, *n* = 74) levels and (**D**) variation in procalcitonin (delta-PCT, *n* = 21). Data are represented as the median with range. Mann–Whitney U, Kruskal–Wallis, and Dunn’s multiple-comparison tests and Spearman rank correlation were performed. ** *p* < 0.01 and *** *p* < 0.001. ns, non-significant.

Subsequently, we compared neutrophil profiles in patients who required ICU care at any point during their hospitalization (ICU) with patients who did not require ICU care during their hospitalization (non-ICU). The initial neutrophil counts in the two groups were similar, but from day 5, the number of neutrophils was dynamic and able to differentiate the ICU from the non-ICU group. The neutrophil counts were significantly elevated in patients requiring ICU department care compared to non-ICU patients (*p* < 0.0001), and it increased significantly during hospitalization (*p* = 0.016) (Figure 3A). Further quantification revealed that the changes in neutrophil counts over the time course (delta-neutrophil: defined as changes in neutrophil counts between day 0 and day 5 of admission) were significantly different between the ICU and non-ICU patients (*p* = 0.0021) (Figure 3B). Both CRP and procalcitonin showed strong positive associations with the changes seen in the neutrophil counts (r = 0.44, *p* < 0.001 and r = 0.60, *p* = 0.003, respectively; Figure 3C,D).

### 3.4. Neutrophil Counts on Day 5 Appeared to Be Excellent Predictors of Complications During Hospitalization

**Figure 4:**
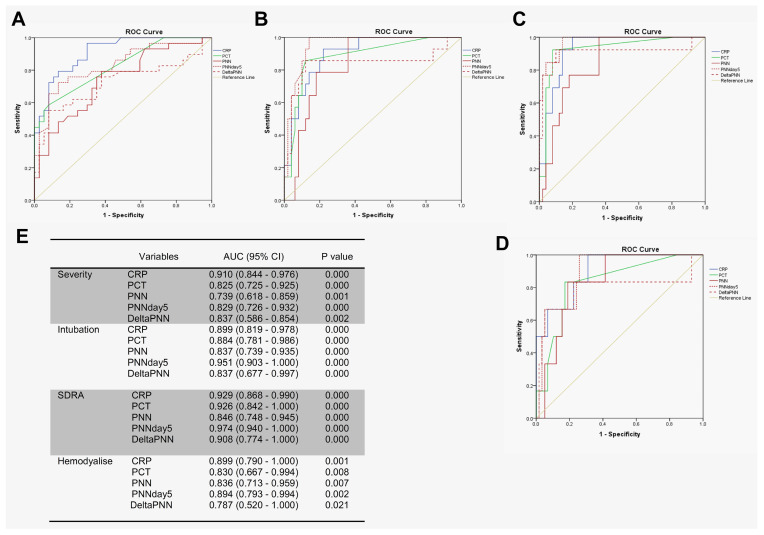
**PNN on day 5 predicted complications during hospitalization.** ROC analyses using day 0 of PNN, CRP, PCT, delta-PNN, and the PNN on day 5 were performed: (**A**) mild–moderate (*n* = 37) and severe–critical (*n* = 29); (**B**) intubation (*n* = 14) or not (*n* = 50); (**C**) ARDS (*n* = 13) or not (*n* = 50); (**D**) hemodialysis (*n* = 6) or not (*n* = 58); and (**E**) area under the curve (AUC) with 95% confidence interval (CI).

We tested the prognostic utility of neutrophil counts in determining the need for advanced respiratory support for patients with COVID-19, using receiver operating characteristic (ROC) curve analyses involving patients with mild–moderate versus severe–critical disease. In particular, we first compared the predictive values of disease severity for CRP, PCT, and neutrophils. Interestingly, CRP, PCT, neutrophil counts (on day 0), neutrophil counts (on day 5), and delta-neutrophils (day 0 to day 5) were all predictive of a need for advanced respiratory support. However, the area under the curve (AUC) for CRP was higher than for the other features (Figure 4A,E).For the prediction of intubation and ARDS, the AUCs of neutrophils on day 5 were 0.951 and 0.974 compared to 0.899 and 0.929 for CRP, respectively (Figure 4B,C,E). For hemodialysis prediction, the AUC of neutrophils on day 5 was in close similarity to that of CRP (0.894 for neutrophils on day 5 compared to 0.899 for CRP) (Figure 4D,E).

**Figure 5:**
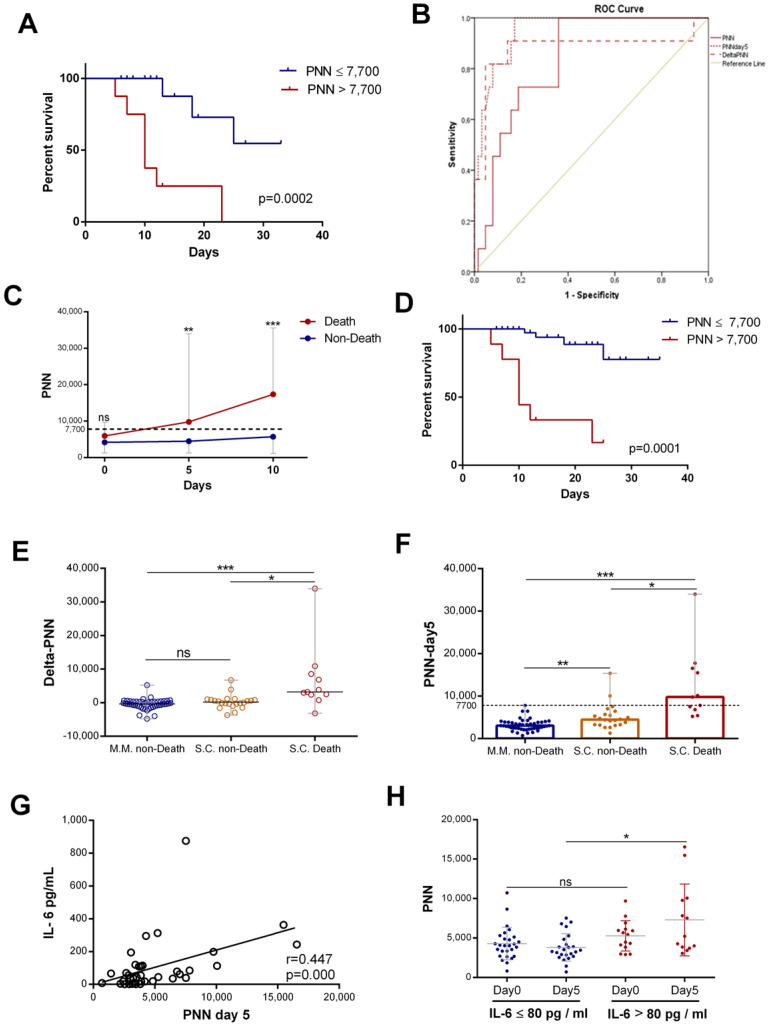
**Neutrophil counts on day 5 associated with IL-6 levels and patient mortality**: (**A**) survival curves for COVID 19 with normal ((≤7500), *n* = 49) and high neutrophil counts ((>7500), *n* = 9) on day 5; (**B**) ROC analysis using PNN on day 0, delta-PNN, and PNN on day 5 performed for death (*n* = 11) or not (*n* = 53); (**C**) evolution of PNN (*n* = day 0 (13 vs. 23), day 5 (11 vs. 22), and day 10 (9 vs. 14)) count during the first 10 days of hospitalization in patients grouped into death or non-death categories, respectively (represented as the median with range); (**D**) survival curves for severe–critical patients with normal ((≤7500), *n* = 29) and high neutrophil counts ((>7500), *n* = 8) on day 5; (**E**) variation in PNN (delta-PNN = PNN-day 5 − PNN-day 0) in patients with mild disease who survived (MM, non-death) (*n* = 47), those with severe disease who also survived (SC, non-death) (*n* = 22), and those with severe disease who did not survive(SC, death) (*n* = 11); (**F**) PNN count on day 5 in patients with mild disease who survived (MM, non-death) (*n* = 49), those with severe disease who also survived (SC, non-death) (*n* = 22), and those with severe disease who did not survive (SC, death) (*n* = 11) (represented as the median with range); (**G**) correlation of variation in PNN on day 5 and initial IL-6 (*n* = 44) levels; and (**H**) PNN count in patients with IL-6 levels >80 pg/mL on day 0 (*n* = 15) and on day 5 (*n* = 13) or <80 pg/mL on day 0 (*n* = 27) and on day 5 (*n* = 24) (represented as the mean and standard deviation). Kruskal–Wallis and Dunn’s multiple-comparison tests were performed. * *p*< 0.05, ** *p* <0.01 and *** *p*< 0.001. ns, non-significant.

The neutrophil counts were associated with mortality in hospitalized patients with COVID-19. Patients were stratified into two groups, those with normal (≤7500/mm^3^) and high neutrophil counts (>7500/mm^3^). Kaplan–Meier curves for all patients were drawn to study the association of neutrophil counts on day 5 with COVID-19 mortality. The results showed that a rise in neutrophils was statistically associated with COVID-19 mortality (Figure 5A). Then, we assessed the predictive value of neutrophil counts (PNNs) at admission, that of changes in neutrophils from day 0 to day 5 (delta-PNN), and that of neutrophil counts on day 5 (PNN-day 5) for all patients with COVID-19. All three were predictive of mortality, though the area under the curve (AUC (95% CI)) for PNN-day 5 was greater than for the other parameters. The AUC for neutrophils on day 5 was 0.951 (0.903–0.999) compared to 0.881(0.723–1) and 0.834 (0.733–0.934) for delta-PNN and neutrophils at admission, respectively (Figure 5B). Subsequently, we split the severe–critical patients into two groups (death and non-death). Interestingly, we found that patients with normal neutrophil counts survived, and on the other hand, those with a gradual increase in neutrophil counts exceeding the normality threshold died (Figure 5C). The survival curve of patients with severe disease revealed that the neutrophil count on day 5 was a statistically significant risk, which was associated with mortality in patients with severe COVID-19 (Figure 5D). These results showed that, in patients with severe disease, two subgroups could be distinguished based on the number of neutrophils. Surviving patients were characterized by normal neutrophil counts during the first 10 days of hospitalization, while non-survivors were characterized by an abnormal increase in the neutrophil number (>7700/mm^3^) from day 5.

With the aim of extending this observation to all patients, we studied the variation in neutrophil counts according to disease severity and mortality in all patients. The results showed that, in patients with mild disease (MM) and in survivors with severe disease, there was no statistically significant difference between neutrophil count variations (delta-PNN) (Figure 5E) and the count on day 5. The neutrophil counts of these two groups of patients were normal (Figure 5F). In contrast, patients who did not survive exhibited significantly elevated neutrophil counts on day 5 relative to the control (Figure 5F). These results suggested that dynamic neutrophil trends in patients with COVID-19 and their rates on day 5 were predictive of mortality, and this was independent of clinical classification.

To confirm these results, we carried out a logistic regression analysis with the enter or forward selection method to identify risk factors for mortality and ARDS. In this analysis, there were 79 patients with COVID-19 whose neutrophil level on day 5 had been recorded, alongside their survival status. A binary logistic regression was performed on the neutrophil count(PNN), neutrophil count on day 5 (PNN-day 5), neutrophil-to-lymphocyte ratio (NLR), C-reactive protein (CRP), procalcitonin (PCT), creatinine, hemoglobin (HB), and age. For selecting relevant classification predictors, we used the forward stepwise algorithm based on Wald’s test. The algorithm identified that neutrophil counts on day 5 were the risk factor that best predicted mortality among patients with COVID-19 (Table 2). Thus, our algorithm employed neutrophil counts on day 5 as the first variable for predicting mortality and then either the CRP or the PCT.

We also performed binary logistic regression with PNN-day 5, which was stratified as an explanatory/predictor variable. We distinguished two possible variables as dependent variables—“death” or “ARDS”. In both cases, we found that PNN-day 5 > 7.7 × 10^9^/L discriminated well between the two mortality classes “yes/no” and the two ARDS groups “yes/no” for patients with COVID-19 (Table 3). The pooled logistic regression model for each case was the following:

$$Logit\;(P(mortality=1/PNNday5)\;)=-2.72+3.57\times {ind}_{PNN-day5>7.7(109\backslash L)}$$

$$Logit\;(P(ARDS=1/PNNday5)\;)=-2.46+3.85\times {ind}_{PNN-day5>7.7(109\backslash L)}$$

We also knew the following:

$${ind}_{PNN-day5>7.7\times 109\backslash L}=\left\{\begin{array}{cc} 1 & \;\;\;if\;\;PNN-day5>7.7(109\backslash L)\;\\ 0 & else \end{array}\right.$$

The neutrophil count on day 5 was found to be an independent predictor factor for mortality and ARDS. Neutrophil counts on day 5 could therefore help clinicians identify patients with COVID-19 who have a poor prognosis and provide them with suitable care.

Furthermore, in this study, we investigated the relationship between IL-6 levels and neutrophil level increase. Interestingly, the IL-6 levels showed a positive correlation with neutrophil counts on day 5 (r = 0.447, *p* = 0.000) (Figure 5G). Then, we compared neutrophil counts in patients with high or low initial IL-6 levels. We found that the levels of neutrophils on day 5 were statistically more elevated in patients with high initial IL-6 levels than in patients with low IL-6 levels (Figure 5H).

Our study suggested that increased neutrophil counts might be associated with a previous increase in IL-6 levels in patients with COVID-19.

## 4. Discussion

This study of patients presenting with COVID-19 provided three key findings. First, the neutrophil count at patient admission was higher in severe cases compared to non-severe disease. Remarkably, as the disease progressed, the neutrophil counts increased among patients with severe disease and those requiring ICU care at any point during their hospital stay. This finding suggested that neutrophil counts are dynamic in patients with severe COVID-19. At the same time, we found that an increase in neutrophils had a remarkably close association with an increase in inflammatory biomarkers (CRP and procalcitonin). The results, similar to those in the literature, indicated that neutrophil counts in patients with severe disease were higher than in patients with non-severe disease and that these counts increased especially among severe cases [14]. Another study also found that patients with severe COVID-19 had higher NLR levels, compared to the non-severe group (*p* < 0.001), and that in the severe ICU group, patients had the highest neutrophil counts and NLR relative to the other groups (severe non-ICU and non-severe group) [8]. In another report, Wang et al. found that there were numerous differences in laboratory findings between patients admitted to the ICU and those not admitted to the ICU, including higher white blood cell and neutrophil counts [15]. Neutrophils are the most abundant immune cells in human blood [16]. These participate in antiviral defense through interactions with other populations of immune cells and the release of cytokines and neutrophile extracellular traps (NETs) [17,18]. The latter are known to immobilize and degrade viruses [19]. Studies have shown an elevated rate of NETs in patients with COVID-19, and their increase is correlated with an increased severity of the disease [20,21,22]. Although neutrophils may have a protective role, their prolonged activation might have adverse effects on the lungs and lead to pneumonia and/or ARDS [6,23]. Neutrophils are present in many lung diseases associated with ARDS, such as influenza virus infections and SARS-CoV-1 [24]. In addition, persistently activated neutrophils contribute to the maintenance of the inflammatory state in the lungs through the release of cytokines in MERS and SARS-CoV-1 infections [25]. Recently, neutrophilia has been described as an indicator of severe respiratory symptoms and a poor prognosis in patients with COVID-19 [26,27,28], and a high ratio of neutrophils to CD4+ lymphocytes (NCD4LR) indicates poorer immune function and prolonged viral clearance in this patients population [29].

Several studies have reported that the NLR predicts severe disease in patients with COVID-19 [26,30,31] and is strongly related to the aggravation of ARDS [32]. A study reported that the area under the ROC curve for moderate–severe ARDS was 0.749 for NLR [33]. Neutrophils can be measured during routine hematology. Previous studies have shown that an increase in neutrophil counts is associated with clinical deterioration for patients with COVID-19 and that neutrophil counts upon patient admission can predict ARDS. However, it remains unclear to what extent the significance of neutrophil counts would predict COVID-19-related complications or when this prediction is better. The second key finding of our study was that the neutrophil counts on day 5 proved to be excellent predictors of complications during hospitalization. We compared the predictive values of CRP upon admission, PCT levels, neutrophil counts (day 0), changes in neutrophils from day 0 to day 5, and the neutrophil counts on day 5. For the prediction of intubation and ARDS, the values of the area under the curve (AUC) for neutrophils on day 5 were 0.951 and 0.974 compared to 0.899 and 0.929 for CRP, respectively. For the prediction of hemodialysis and severity, the AUC values for PNN-day 5 were 0.894 and 0.829 compared to 0.899 and 0.910 for CRP, respectively. Thus, neutrophil counts on day 5, rather than at admission, were more closely associated with clinical deterioration.

Third, our results suggested that dynamic trends in neutrophils in patients with COVID-19 and their rates on day 5 were predictive of mortality, and this was independent of clinical classification. However, neutrophil dynamics in patients with COVID-19 have not been studied before day 5. It has been reported that the neutrophil-to-albumin ratio (NAR) can be a simple marker for predicting mortality in patients with COVID-19 [34]. For instance, multivariate analysis has shown that an increased NLR was predictive of mortality [35], while high NLR levels upon admission have been associated with severe COVID-19 and mortality [36,37].

It has been reported that IL-6 induces neutrophilia [38]. IL-6 also drives neutrophil-mediated pulmonary inflammation [39]. On the other hand, a study has shown that the risk of respiratory failure for patients with COVID-19 with IL-6 levels > 80 pg/mL was 22 times higher compared to patients with lower IL-6 levels [40]. Therefore, we analyzed neutrophil levels on day 5 in two groups of patients (patients with initial IL-6 levels above or below 80 pg/mL). Interestingly, the results showed a positive correlation between neutrophil counts on day 5 and IL-6. Furthermore, neutrophil levels on day 5 were statistically more elevated in patients with initial levels of IL-6 > 80 pg/mL compared to those of patients with levels ≤ 80 pg/mL. These data suggest that the observed increase in neutrophils might be due to a previous increase in IL-6 levels in patients with COVID-19.

## 5. Limitations

This study has a few limitations, the most important being the relatively low number of included patients. This can be explained by the relatively low number of patients admitted to our unit in the study period and by the fact that this study took into account the vaccination status of patients, ensuring that only unvaccinated patients would be studied, to exclude the potential effect of vaccination on cellular immunity. Second, our study strongly suggests that neutrophil counts on day 5 (PNN-day 5) are strong predictors of complications and mortality. However, the dynamic changes occurring in the number of immune cells between admission and the fifth day have not been studied. The patient admission protocol specific to our institution allowed us to carry out analyses upon admission and at 5-day intervals during follow-up. It was not possible to engage patients without their consent in additional analyses as per the standard protocol of our hospital center. Furthermore, this study did not consider validation methods such as cross-validation for type I error correction. Future large-scale studies to confirm our results are needed, and we plan to conduct analyses after 6 h, 24 h, 48 h, and 5 days of admission to study the dynamic changes in neutrophil counts that occur between admission and day 5 of patient hospitalization for early-stage predictions.

## 6. Conclusions

In conclusion, in this retrospective study of patients with COVID-19, neutrophil counts on day 5, rather than at admission, proved to be excellent predictors of complications during hospitalization. This parameter could be used to effectively predict potential mortality independently of usual clinical classifications. These results could be used to facilitate patient stratification management.

## Figures and Tables

**Table 1:** Characteristics of the study population according to disease severity.

Clinical Variables	Mild–Moderate (*n* = 83)	Severe–Critical (*n* = 36)	*p*-Value
Age, mean (SD), y	44 (16)	66 (11)	0.003
Male, no. (%)	37 (44.6)	29 (80.6)	0.000
Clinical symptoms, no. (%)			
Asthenia	2 (2.4)	20 (55.6)	0.000
Fever	30 (36.1)	22 (68.8)	0.002
Headache	13 (15.7)	5 (15.6)	0.996
Gough	34 (41)	17 (53.1)	0.239
Dyspnea	15 (18.1)	22 (66.7)	0.000
Vomiting	16 (19.5)	6 (18.2)	0.87
Diarrhea	17 (20.5)	10 (31.2)	0.22
Ageusia	16 (19.5)	5 (15.6)	0.63
Past medical history, no. (%)			
Asthma	5 (6)	5 (13.9)	0.155
Cardiac disease	3 (3.6)	11 (30.6)	0.000
Diabetes	7 (8.4)	10 (27.8)	0.006
Renal failure	0	2 (5.6)	0.03
Autoimmune disease	3 (3.6)	3 (8.3)	0.28
Smoking	4 (4.8)	2 (5.6)	0.866
Complications			
ARDS	0	13 (40.6)	0.000
Arterial hypertension	0	7 (23.3)	0.000
Hemodialysis	0	8 (24.2)	0.000
Mortality	0	13 (39.4)	0.000

**Table 2:** **Regression analysis of mortality risk factors for patients with COVID-19.** Data are represented as pooled logistic regressions. Abbreviations: NLR, neutrophil-to-lymphocyte ratio; CRP, C-reactive protein; PCT, procalcitonin; HB, hemoglobin; OR, odds ratio; and CI, confidence interval.

	Mortality	
	OR (95% CI)	*p*-Value
Neutrophils on day 5	1.001 (1.00–1.001)	0.020
Neutrophils	0.999 (0.998–1.001)	0.360
NLR	0.998 (0.992–1.004)	0.525
CRP	1.010 (0.985–1.036)	0.432
PCT	4.655 (0.044–367.372)	0.519
HB	1.707 (0.650–4.481)	0.278
Creatinine	0.958 (0.671–1.366)	0.811
Age	1.021 (0.944–1.104)	0.609

**Table 3:** **Pooled regression analysis of mortality and ARDS risk factors for PNN-day 5.** Data are represented as pooled logistic regressions. Abbreviations: PNN-day 5, neutrophil count on day 5; ARDS, acute respiratory distress syndrome; B, regression coefficient; SE, standard error; OR, odds ratio; and CI, confidence interval.

Variable	Parameter	B	SE	*p*	OR (95% CI)
Mortality	PNN-day 5	3.572	0.862	0.000	35.583 (6.573–192.644)
ARDS	PNN-day 5	3.854	0.918	0.000	47.200 (7.814–285.092)

## Data Availability

The datasets analyzed during the current study are available from the corresponding author upon reasonable request.

## Abbreviations

The following abbreviations are used in this manuscript:

ARDS	Acute respiratory distress syndrome
AUC	Area under the curve
CRP	C-reactive protein
CI	Confidence interval
COVID-19	Coronavirus disease 2019
NETs	Extracellular neutrophils traps
HB	Hemoglobin
ICU	Intensive care unit
IL-6	Interleukin 6
M.M	Mild–moderate
PNNs	Neutrophils
PNN-day 5	Neutrophils on day 5
NAR	Neutrophil-to-albumin ratio
NLR	Neutrophil-to-lymphocyte ratio
OR	Odds ratio
PCT	Procalcitonin
ROC	Receiver operating characteristic
SARS-CoV-2	Severe Acute Respiratory Syndrome Coronavirus 2
S.C	Severe–critical
SE	Standard error
WBC	White blood cell
WHO	World Health Organization
